# The politics of the surgical mask: Challenging the biomedical episteme during a pandemic

**DOI:** 10.1111/jep.13590

**Published:** 2021-06-16

**Authors:** Shane Neilson

**Affiliations:** ^1^ Department of Family Medicine McMaster University, Waterloo Regional Campus Ontario Canada; ^2^ Department of English University of Ottawa Ontario Canada

**Keywords:** COVID‐19 pandemic, humanity, philosophy of medicine

## Abstract

COVID‐19 has seen politicians use a selective ‘science’ to justify restrictions on mobility and association, to mandate the wearing of face masks, and to close public infrastructure. There seems to be no role for health humanities scholars as yet, but perhaps there should be. This paper considers the fate of a health humanities article on surgical mask use that was published in a biomedical journal in 2016. This article, which did not operate from within the biomedical episteme but which was in conversation with the episteme, was misappropriated on both sides of the political spectrum to justify personal beliefs around mask use in the pandemic. This mistaken misappropriation is not only evidence of the utility of the common ground shared between biomedicine and the health humanities, it is also evidence of the possibilities inherent in a future interdisciplinary involving biomedicine and the health humanities.

## 1 INTRODUCTION

The recently published *Routledge Handbook of the Medical Humanities* states that it will look at its subject as ‘network and system, therapeutic, provocation, forms of resistance, a way of reconceptualising the medical curriculum, concerned with performance and narrative, mediated by artists as diagnosticians of culture through public engagement’.^1^ From this list one can easily abstract the general, simple, and necessary purpose of the medical humanities, which is to critique and counterbalance the cultural primacy of biomedicine – or, as Paul Crawford puts things in this introduction to a different text, the *Routledge Handbook of the Health Humanities*, to act as a kind of ‘creative public health’.^2^ * In this era in which biomedicine governs the way most people think about health, a basic function of any medical humanities work is to resist that dominance by challenging the episteme. Defining my terms for a moment, and relying on the work of Norman Sartorius as I do, I intend ‘health’ to mean a state of balance, an equilibrium that an individual has established within himself and between himself and his social and physical environment^3,p. 663^ and not the historical one in which ‘health is the absence of any disease or impairment’ because the former is in excess of biomedical techniques of measurement whereas the latter is entirely adjudicated by biomedicine. Or as Sartorius explains, ‘If health is defined as the absence of disease, the medical profession is the one that can declare an individual healthy’.^3,p. 662^ The medical humanities put forward an alternative set of methods and a different vision for what it means to be ‘healthy’, but the key overarching intervention remains that the field, by virtue of its mere existence, insists on contributing to a definition of what being ‘healthy’ means. In this article, I will consider a purpose of medical humanities scholarship – to resist biomedicine – as emphasized by the life‐and‐death stakes of COVID‐19. I provide a ‘case’ in which my health humanities work is deliberately misread for disparate political agendas. This paper considers the uptake of my article from 2016 on Twitter on behalf of anti‐maskers and pro‐maskers, showing that my article is misread by both sides according to a credentialism that can yet be mobilized for the benefit of the medical humanities. I will further argue that non‐biomedical cultural logics, when properly framed, have a practical and yet non‐instrumental application both in merely being themselves – an alternative to biomedicine is useful as a means of understanding ‘health’ alone. In short, beyond the more complex tack any scholarly inquiry may take regarding health, pandemics make humanities and social sciences scholarship matter, for all such scholarship contributes to the urgent project of counterbalancing biomedical primacy. Perhaps the health humanities in particular have a unique role to play as compared to other fields because it exists in a unique relation with biomedicine itself.

That the humanities and social sciences are, relative to STEM disciplines, under threat in the contemporary era of university corporatization is accepted as common knowledge. The privileging of STEM within universities and in the Canadian economy is part of a broader cultural logic that has certain predictable effects. A related (and probably sponsoring) phenomenon is the dominance of biomedicine when it comes to societal imaginings of what it means to be healthy. It is important to recognize that biomedicine is ‘a framework, a set of philosophical commitments, a global institution woven into Western culture and its power dynamics, and more. Biomedicine is the umbrella theoretical framework for most health science and health technology work done in academic and government settings’.^4^ Paraphrasing the work of Nancy Krieger, *The Stanford Encyclopedia of Philosophy* goes on to offer a useful definition of biomedicine as cohering around three key tenets: (a) ‘the domain of disease and its causes is restricted to solely biological, chemical, and physical phenomena’; (b) there is ‘an emphasis on laboratory research and technology and, as translated to health research, a discounting of research questions that cannot be studied by randomized clinical trials’; and (c) biomedicine features ‘an embrace of “reductionism,” a philosophical and methodological stance . . . that holds that phenomena are best explained by the properties of their parts’.^4^

In ‘On Evidence and evidence‐based medicine: Lessons from the philosophy of science’, an article that unpacks the reasons behind the ‘shortcomings of biomedicine for properly addressing women's health needs, as articulated by feminist scholars and allies of the women's health movement’,^5,p. 2627^ Feminist epistemologist Maya Goldenberg points out that biomedicine is largely an effort to manage the unruly social world in which medicine is practiced via objective scientific procedure, the movement appears to be the latest expression of ‘scientism’, modernity's rationalist dream that science can produce the knowledge required to emancipate us from scarcity, ignorance, and error. That society has been medicalized is a phenomenon well‐documented by scholars like Ivan Illych,^6^ Thomas Szasz,^7^ and Peter Conrad.^8^ However, what is germane about that fact for my purposes is that such efforts tend to disguise political interests in the authority of so‐called ‘scientific evidence’. As Goldenberg explains, the configuration of policy considerations and clinical standards into questions of evidence conveniently transform normative questions into technical ones.^5,p. 2630^ These technical questions have vast implications in society, for what we are arguing about at the level of a technique is inevitably also a political question.†

One of the technical questions of great importance in the early stages of the pandemic concerned whether to wear a mask or not in public and private spaces, and it is no surprise that the debate occurred in society on the level of evidence and expert recommendation. It is not the point of this article to unpack the shifting policy decisions and relatively scant evidentiary basis of the mask debate – indeed, some of this work is already done in my original article from 2016, and if you wish to have a more recent update on the still‐contested topic, see Trish Greenhalgh's ‘Face masks for the public during the COVID‐19 crisis’^9^ that spurred a flurry of responses and counter‐responses in recent medical literature – but rather to examine the political life such matters adopt when dancing around the pole of biomedicine.

Because lockdown circumscribes the social lives of citizens largely to the space of their immediate confinement, our thinking as social beings was effectively put into quarantine because our bodies are placed in quarantine and/or social distancing. In consequence, fear comes to masquerade as ‘best evidence’ or, more often, as the interpretation of said evidence – yet the evidence is problematic, as are the interpretations of the evidence.‡ Biomedicine becomes bulwark against, but also a projection of, fear. The question of to mask or not to mask, or lockdown or not to lockdown,§ was run through this same process, of a confusing and contradictory evidentiary debate by scientists and, as will be seen, by people at home capitalizing on different findings and interpretations in order to justify their political orientation, broadcasting same on social media and via email correspondence. That broadcast is largely fear‐driven.

Individuals with pre‐existing political differences will use biomedical information to accomplish disparate political goals, abetted by the nature of data, for it is not in the nature of biomedicine to be an arbiter of anything except a numeric truth. The cultural power of biomedicine will be co‐opted to serve atavistic political agendas and some of the tools required to inform the corrective – I use the word with all its baggage – are the disciplines of the humanities and social sciences in general, and perhaps the field of the health humanities in particular. For our own good, we must temper biomedicine with some alternative ‘common sense’; specifically, we could bring out the inherent qualities of the qualitative approach and permit science to speak in a different way.

## 2 CURIOUSER AND CURIOUSER: THE CULTURAL FATE OF A HEALTH HUMANITIES ARTICLE PUBLISHED IN A BIOMEDICAL JOURNAL

In his introduction to the *Routledge Companion to Health Humanities*, Paul Crawford writes that the ‘health humanities adopt an interdisciplinary, inclusive, applied, democratizing, and activist approach to the arts and humanities in informing and transforming health care, health, and well‐being’.^2,p. 3^ Though the number of ways the health humanities can disrupt biomedical hegemony are numerous, the one I will develop in this article is inverse to that which Crawford encourages: rather than artisfy medicine, I wish to emphasize the contact point the health humanities already has with biomedicine. Though the fields are homologous in terms of health, they are irreconcilable in terms of their respective ‘tenets’, and in accentuating the common ground both fields have, one can see – speaking metaphorically – a bridge to alternative possibilities. Reveal biomedicine as an important but not total contributor to a larger concept of health and biomedicine's totalizing disguise of ‘common sense’ is revealed as a normative preference that poorly serves the non‐normative, but which poorly serves normative demographics also. In the context of this article, itself reflective of the rise of the health humanities and a cultural phenomenon in which health humanities research is increasingly placed in conversation with biomedicine, we are positioned to critique the ‘common sense’ aspect of biomedicine when it comes to pandemic response.

‘Common sense’ is a dead metaphor to explain a dead cultural logic, a concretization of thought such that norms are assumed. The humanities and the social sciences reveal common sense's normativity, and when that exposure is done effectively enough, the humanities and social sciences face pushback. It is exactly this ratifying pushback that serves as the ‘best evidence’ scholars in the humanities and social sciences have concerning their contributions to public life. Often, the problematic inherent to the feedback is misapplication of biomedical methodologies to humanities methodologies, a misapplication encouraged by the dominance of the biomedical episteme in the culture. I will now develop such an instance of the public reception of health humanities scholarship concerning pandemics as compelling evidence that health humanities work is worth doing and might function as a path to redistributing cultural power. The vigorous life a Canadian's modest analysis of the use of surgical masks in non‐pandemic conditions has taken online shows how health humanities research possesses cultural capital, and though the deliberate misreadings of the article are predictable based on the ethos of social media, the common ground shared between these misreadings suggests that there might be an opportunity to recalibrate cultural preference away from biomedicine's reductive view of health and towards a more inclusive one. The stakes are simple and important. If health humanities research is a football that different political actors wish to run with, what might happen if health humanities scholars were given the ability to take back the football? What would happen if biomedicine became less a refutational or dismissive force when it came to health humanities research and were instead used to encourage interested parties to thoughtfully consider a different system of knowledge?

In 2016, I published an article titled ‘The Surgical Mask is a Bad Fit for Risk Reduction’ in the *Canadian Medical Association Journal*.^11^ This article appeared in a new section of the journal called ‘Medicine & Society’ that is dedicated to publishing on biomedical topics from the perspective of social sciences and humanities scholarship. Nothing like that section had ever appeared in a flagship medical journal before (though plenty of work appeared in dedicated health humanities journals for decades; the kind of work was not new, but the hybridization of venue was.) Suddenly, doctors in Canada might read McGill‐based health architecturist Annemarie Adams on how Canadian hospital design has transformed (‘Canadian hospital architecture: how we got here’), with its breathtaking two‐way street observation that ‘Our 11 architecture schools are only beginning to teach basic health care design; and our medical students don't learn about architecture’.¶ ^12^ It is heretical to imply that health care in Canada could be improved if medical students learned more about architecture, but such heresy is conducted in the ‘Medicine and Society’ column with regularity. Or Canadian physicians might read the University of Alberta's disability studies scholar Heidi Janz on ableism in medicine ‘Ableism: the undiagnosed malady afflicting medicine’, in which the unquestioned ‘good’ of medicine itself is questioned in the opening paragraph:

Medicine has traditionally been viewed as a benevolent discipline in which every human life is valued equally, without any form of prejudice or discrimination. Although this may remain the ideal to which medicine aspires, the reality is that, as individuals, medical professionals are not immune to the influence of dominant societal understandings of, and attitudes toward, individuals and groups of people deemed to be ‘others’.^13^

The appearance of health humanities scholarship critiquing biomedicine amidst what is Canada's flagship biomedical purview was a remarkable development, but one with unintended consequences for the field of the health humanities.

I close read the use of surgical masks in movies as they connected with other circulating public documents like pandemic response guidelines from the Public Health Agency of Canada and Ulrich Beck's risk society theory taken from *Risk society: towards a new modernity*.^14^ Demonstrating a competence with biomedicine itself – for a lack of competence demonstrated in this field is always the first line of an attack from a biomedically informed practitioner – I provided a brief but pertinent summary of relevant articles concerning mask use and their effectiveness in pandemics.** It is at this biomedical contact point that I created the future conditions for biomedicine's inevitable retaliation. In my article, as part of providing basic authentication inherent to systematically consulting and reviewing a literature, I wrote:

The birth of the mask came from the realization that surgical wounds need protection from the droplets released in the breath of surgeons. The technology was applied outside the operating room in an effort to control the spread of infectious epidemics. In the 1919 influenza pandemic, masks *were* available and *were* dispensed to populations, but they had no impact on the epidemic curve. At the time, it was unknown that the influenza organism is nanoscopic and can theoretically penetrate the surgical mask barrier. As recently as 2010, the U.S. National Academy of Sciences declared that, in the community setting, ‘face masks are not designed or certified to protect the wearer from exposure to respiratory hazards’. A number of studies have shown the inefficacy of the surgical mask in household settings to prevent transmission of the influenza virus . . .^11^

Via this short summary, I engaged with biomedicine on its terms so that there could be a productive conversation between fields. I brought biomedicine's data into the realm of the humanities so that both could be used, together, to make a new kind of argument, this being the work and unique contribution of the health humanities.

Four years later, COVID happened, boosting the article into the top 5% viewed on record in the Altmetric data service. Because there was not a lot of useful biomedical information available about pandemics like the current deadly one (hence the initial erroneous recommendations by our public health officials) and more specifically because there was not a lot of information available about the use of surgical masks in pandemics (this is changing), COVID made the issue of mask use early on in the pandemic a huge concern. The choice of whether to wear one or not took on a political dimension. By extension, my article took on a political dimension, too, and not the one I had intended, owing, again, to the unique relation biomedicine has with the health humanities.

Before I proceed to unpack the COVID‐instigated selection of citations and mentions of my article in the public realm, however, I set this frame: what follows is what I call the *false common ground* between health humanities and public policy. The false common ground is one that operates as ‘evidence’ with concern to ‘health’. As will be seen, this false common ground can also be thought of as a misapplication of biomedical methodologies to health humanities findings. In this misapplication, the cultural logics of one field are misappropriated for a political purpose as it concerns human health. In other words, what follows is the result of a collision between misapplications of scholarship and politics as both concern human health.

On 16 April 2020, I received the first piece of pandemic‐related correspondence about the article. I wish I could provide the entirety of it as one long block quote, or even quote from a part of it, but I am wary of doing so because I would need to ask for permission for the right of quotation, this being a private missive. Based on how my article has come to be used since, I do not want to offer any encouragement for further misuse, nor do I want to provide a quote that could be further misused on social media. My first emailer reasonably pointed out that though many States mandated mask use, based on his own observations in stores he felt that shoppers and staff were touching products and their own masks so frequently that his interpretation of my article – that surgical masks might be a falsely reassuring symbol of protection – was true. Moreover, he added that COVID‐19 was a pretense for an attack on liberty, privacy, and free enterprise due to strategically exaggerated fear by government. Rather than considering how masks might have unintended negative consequences as symbols of fear, my correspondent thought that the article was proof that surgical masks were misguided protective measures in a pandemic and that my article should inform public policy. The reader misinterpreted my article as scientific, as biomedical.

On 5 May 2020, I received another email, but this time from a critic who asked what my thoughts were concerning the use of my article online by unnamed others to discourage the wearing of masks during COVID‐19. By this point, more thinking had been done about the role of masks and a consensus was forming that though masks might not protect the wearer from contracting COVID, they might reduce the spread of viral particles to others from infected wearers. Again, this correspondent was uninterested in Ulrich Beck, failing to mention the surgical mask as symbol; their interest in the article was also based on a reductive reading dependent on mistaking the article for a legitimate trial of mask use during a pandemic.

Now actively wondering why my article was receiving *this* kind of feedback, I began to do research myself. I discovered a letter published online that was directed to the city council of Montgomery, Alabama and that republished the text of my article. The letter contains the following paragraph: ‘My wife works in the neonatal intensive care unit and is required to wear a mask for 12 hours shifts. She has symptoms of hypoxia from too much CO_2_ and she finds herself touching her face more often to readjust her mask and get small breaks with fresh air’.^15^ Hypoxia from surgical mask use is not possible, but the contention became such an oddly successful disinformation meme that dozens of disabled and medical professionals took to Facebook, Twitter, and Instagram to debunk it with live demonstrations.

Despite widespread performances on social media demonstrating no oxygen desaturation with mask use, the article ‘Wear Your Face Mask and Savor your CO_2_ Breath’ was published May on *crossradio1.org*.^16^ The web article expands upon the nonsensical idea that surgical masks cause people to rebreathe relatively concentrated CO_2_ to deleterious effects. The article uses my *CMAJ* piece as justification for its larger purpose, to discourage mask use, by invoking the fact that his article was ‘peer reviewed’, that it is found on the ‘National Institute of Health's website’, and that it is written by an ‘M.D.’^16^ The theme of the article is personal freedom, and a sinister light is thrown upon the machinations of government: ‘Thus, the government's goal to encourage the continued use of facial masks is to reinforce fear so that their own risk management problems can be intensified. This, in pure terms, is nothing short of psychological manipulation and it has nothing to do with health or keeping people safe’^16^ The use of the close quote at the end is particularly misleading because the implication is that it is *my* words which are being transcribed, but I did not write this paranoid vision.

**Figure 1:**
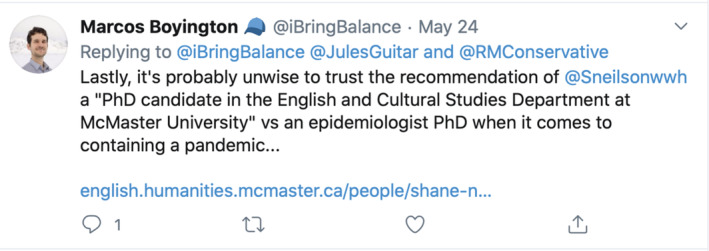
Screenshot of a tweet from Marcos Boyington that tries to erroneously reject a health humanities paper about surgical masks for biomedical and credentialist reasons

**Figure 2:**
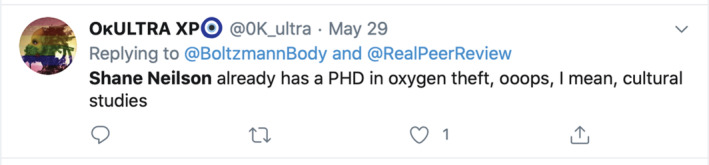
Screenshot of a tweet from an anonymous account that derides a health humanities paper about surgical masks on the basis of its author's identity as a PhD student in “cultural studies”

Twitter soon found me, and in a way that I want to bring forward a different manifestation of ‘false common ground’ between the health humanities and the practical adoption of scholarship in public life. Whereas ‘The Surgical Mask is a Good Fit for Risk Reduction’ was used up this point to support what I interpret as conservative and/or libertarian viewpoints around personal choice (council letter, initial email) or was questioned according to evolving understandings of transmission (second email), what these uses have in common is a reading of my article as an authoritative opinion based in biomedicine. For them, I said ‘Don't wear masks during COVID’, and in each instance my correspondents either mentioned my status as physician or that my article was peer‐reviewed/appearing in a medical database as evidence of authority. For them, the article was valid in some way, either worth agreeing with or worth contesting because it was biomedical. What follows are usages of the article that reflect reading the article as a faulty, invalid biomedical document. The article is rejected because of a lack of authenticity, because its author is not biomedically credentialled; glimpsing these instances brings a true common ground into view.Consider Marcos Boyington, a candidate who was running for the Democratic ticket for the United States Senate in 2020. In an argument about the validity of health mask use with ultraconservative pundit David Horowitz (the account holder of @RMConservative), Marcos opined:

Boyington rejects my ‘recommendation’^17^ (Figure 1) on the grounds of credentialism. Because I am not an epidemiologist, my ‘recommendation’ is untrustworthy. Yet the article is not actually an opinion about whether to wear surgical masks in a pandemic, but is instead a reflection upon the manifestations of risk and how those manifestations are mobilized during times of stress. One might think that those weighing in on the fearful discussion of mask use might keep that fact in mind. In subsequent tweets, Boyington severely criticizes my article's discussion of a paper over 100 years old, picking apart the conclusions I draw. Though the differing interpretations of the article in question are most certainly debatable, at issue here is the biomedical misapplication being made. My piece does not offer a biomedical recommendation, nor is it exhaustively critiquing a biomedical source on biomedicine's terms. To quibble with the biomedicine in a health humanities piece that is trying to use its colleague of biomedicine as one leg to stand on is more than fair, but it is also silly; to narrow in on the biomedical content is to critically analyze a single detail in a larger argument, to the detriment of the argument itself. Yet to discuss metonymy with an interlocutor like Boyington would also be silly. What is not silly is the reductive idea that health is purely biomedical.Twitter offered more commentary from @OkULTRA XP^18^ (Figure 2):

This mash‐up of the bizarre CO_2_ theory along with the co‐proceeding invalidation of a piece of humanities scholarship for offering an opinion that it does not actually offer is the flip side of the Crossradio1.org opinion, with the same strange theme running through. Though one might be inclined to ascribe the different usages of my article as the familiar phenomenon of fake news vended on social media –and more accumulates each week as I update the archive, including a Connecticut‐based chiropractor's Facebook page titled ‘Why an executive order to wear masks?’,^19^ a webpage called *Gweilo Rant*,^20^ and a host of tweets that accuse me of actively spreading the virus^21^ – I yet maintain that there is something to learn from these ‘nonexpert’ usages because of the insights they offer with respect to biomedicine and the health humanities.

Because my article is used for different purposes in the public realm to maintain something that it does not do, and is criticized according to a disciplinary tradition it does not exist in, owing to the cultural dominance of biomedicine, there is an opportunity here to capitalize on the misapplication that is occurring. The error stems from biomedicine's cloaked role as ‘common sense’, leading to the wrong conclusions and mistaken applications used in each of the instances above, though the true common ground of being correctly identified as a humanities scholar who should not be making public policy recommendations predicated on biomedicine is, I suggest, a more productive mistake for my readers thus far to make. Oddly, being sneered at in this context is better than to be used in good faith (no matter how mistaken) as a biomedical expert.

I would like to turn this argument on its head and convert the common ground of biomedicine and the health humanities – that both overlap via the common concern of ‘health’, meaning that this thematic relation can be appropriated in an overruling or dominating fashion for biomedicine's gain, as shown above on both sides of a political spectrum – into more of a concept of interdependence. As I think I have shown in brief, and as feminist epistemologists like Linda Fisher have conclusively shown,^22^ unopposed biomedical thinking as ‘common sense’ has caused a fair amount of harm. During the pandemic, we failed to protect the most vulnerable; poor racialized people are disproportionately getting sick. Though the plight of living in real time is that we would never know otherwise how much good has been done if we had not adopted biomedicine's imperatives, we have to admit that pure biomedicine has abdicated its role as singular driver of good outcomes. Canadians need a fuller picture of how to respond to a pandemic, including the input of social scientists and humanities scholars, not just epidemiologists and physicians. As Mathew Mercuri has explained in an editorial that questions the rhetorical claims of what science is when governments use the term, the pandemic response thus far has been ‘restricted to health fields, in particular virology, immunology, clinical medicine, epidemiology, and public health. . . [s]eemingly absent from the conversation and press briefings were (and are) experts and data from other fields, such as sociology, behavioural science, and economics’.^23^ An irony of the pandemic response has been the marginalization of science within science, not only the marginalization of social sciences and humanities. I expand Mercuri's list to include personnel that could contribute to a ‘creative public health’ as suggested by Paul Crawford.^2^ The interdependence of health humanities and biomedicine is necessary, otherwise we will continue to make the same kinds of mistakes based in ‘common sense’.††

Scholars in the health humanities know that they can always be overruled in general culture if they get their biomedical information incorrect. Demonstrating a general competence with regards to the biomedical literature when generating arguments based on biomedical precepts, traditions, and oppression in humanities articles is always already open to co‐optation when conclusions serve one political agenda or another, just as biomedical information itself is interpreted to justify political agendas daily. Biomedical conclusions seem to have greater currency than the humanities‐based argument that surround perceived simplistic conclusions. But if a pandemic forces information‐starved people, out of fear, to willfully misread a health humanities article in a prestigious biomedical journal, then we obviously are in the midst of a crisis bringing opportunity. The present moment is forcing disparate communities and motivations into relation. There is a willingness to consume information that, thus far, biomedicine has not provided an answer for on its own, that it cannot on its own. Yes, there is an unfortunate premium put on biomedical information, no matter how illegitimate it is, as long as it serves desired political ends. My adventure in biomedical co‐optation and invalidation is evidence that the common ‘theme’ of health in the health humanities and biomedicine requires fully realized visions from both fields in order for the theme itself to be achieved in Canadian society. It is evidence of an opportunity to try to explain what is lacking in our collective imaginations. To have a fuller understanding of health, we need to stop making the category error; it is worthwhile to point out to biomedicalists that they, too, make a category error when considering health only in terms of numbers. As scholars, we need to keep critiquing biomedicine while publishing alongside biomedical papers. In time, the dominant belief that the body‐is‐data may shift slightly towards an understanding that the body‐is‐embodiment. Just as the ‘science is shifting’ in the pandemic – the justification for changing public health recommendations – we need to shift science too, for the good of public health.

## CONFLICT OF INTEREST

Author declares there is no conflict of interest.

## Data Availability

Data sharing not applicable to this article as no datasets were generated or analysed during the current study.

## Endnotes

* There is currently a branding war happening in the field, whether ‘health humanities’ or ‘medical humanities’ is a more appropriate designation for what is ultimately the same kind of work done by the same people. I personally see the rhetorical benefit in preferring ‘health humanities’ insofar as it is a more inclusive term, not privileging the institutions of medicine itself (ie medical education for one) but ultimately I think it a potato/potatoe battle, thus I use the terms interchangeably and on purpose in this article and in life.

† As Maya Goldenberg has pointed out, a key elision at the heart of biomedicine's perpetual success, perhaps especially during a pandemic in which the scientific evidence from which one makes recommendations is limited: ‘By framing the problems of biomedicine as problems of (lack of) evidence exclusively, the assumptions, methods, and practices of scientific medicine go unquestioned’.^5,p. 2630^ The ultimate political maneuver is to obscure the terms of the debate themselves such that we are already operating within the preferred episteme.

‡ What seems like ‘best evidence’ one moment is thrown out the next. Canada's Chief Medical Officer of Health Dr. Teresa Tam has had to walk back the claim that wearing facemasks is not an effective public health measure in pandemics.^10^

§ Canada first imposed lockdowns before it endorsed the use of face coverings.

¶ So much critique of the institution of medicine comes from the health humanities while failing to recognize that reparations require a two‐way street. It is tempting to say that the institution of medicine requires almost reflexive pushback, but more wisely, it is biomedicine that deserves an almost‐reflexive pushback. I distinguish the episteme and practice from the people who do both kind of work.

** Circa 2016. The literature review was completed pre‐covid 19, an event which would throw out a lot of accepted notions.

†† Perhaps the most obvious first intervention concerning masks and the health humanities might be to flip the perception of masks as symbols of fear to, instead, symbols of care and concern for others.

## References

[1] Bleakley A , ed. Routledge Handbook of the Medical Humanities.Abingdon, England: Routledge; 2020.

[2] Crawford P . Introduction: Global health Humanities and the Rise of Creative Public Health. Abingdon, England: Routledge; 2020.

[3] Sartorius N . The meanings of health and its promotion. Croatian Med J. 2006;47(4):662‐664. PMC2080455 16909464

[4] Philosophy of Biomedicine. *Stanford Encyclopedia of Philosophy*; 2020. https://plato.stanford.edu/entries/biomedicine/#WhatBiom.

[5] Goldenberg MJ . On evidence and evidence‐based medicine: Lessons from the philosophy of science. Social Sci Med. 2006;62:2621‐2632. 10.1016/j.socscimed.2005.11.031 16384628

[6] Illych I . Medical Nemesis: The Expropration of Health. New York: Random House; 1976.

[7] Szasz T . The Medicalization of Everyday Life. Selected Essays. Syracuse U P: Syracuse; 2007.

[8] Conrad P . The Medicalization of Society: On the Transformation of Human Conditions into Treatable Disorders. Baltimore: Johns Hopkins U P; 2007.

[9] Greenhalgh T et al. Face masks for the public during the covid‐19 crisis. Br Med J. 2020;369:1435‐1438. 10.1136/bmj.m1435. 32273267

[10] Harris K . Canadians should wear masks as an ‘added layer of protection,’ says Tam. *CBC News Online*; 2020. https://www.cbc.ca/news/politics/masks-covid-19-pandemic-public-health-1.5576895

[11] Neilson S . Surgical masks are not a good fit for risk reduction. Can Med Assoc J. 2016;188:606‐607. 10.1503/cmaj.151236. PMC4868614 27067817

[12] Adams A . Canadian hospital architecture: how we got here. Can Med Assoc J. 2016;188(5):370‐371. 10.1503/cmaj.151233. PMC4786397 26783331

[13] Janz HL . Ableism: the undiagnosed malady afflicting medicine. Can Med Assoc J. 2019;19:E478‐E479. 10.1503/cmaj.180903. PMC6488478 31036612

[14] Beck U . Risk Society: Towards a New Modernity. London: Sage Publications; 1992.

[15] James P . Letter to Montgomery County. 2020. https://www.montgomerycountymd.gov/COUNCIL/Resources/Files/agenda/col/2020/20200421/testimony/item10-PeterJames.pdf

[16] Wood P . *Crossradio.org*. “Wear Your Face Mask and Savour Your CO2 Breath!” 2020. https://crossradio1.org/wear-your-face-mask-and-savor-your-co2-breath/

[17] @ibringbalance. Lastly, it's probably unwise to trust the recommendation of @sneilsonwwh, a “PhD candidate in the English and Cultural Studies Department at McMaster University” vs an epidemiologist PhD when it comes to containing a pandemic. Twitter; 2020, https://twitter.com/iBringBalance/status/1264617986817572865.

[18] @0k_ULTRA. Shane Neilson already has a PhD in oxygen theft, ooops, I mean, cultural studies. Twitter; 2020, https://twitter.com/0K_ultra/status/1266478324437712898

[19] *Shaw Family Chiropractic* Facebook Page. “Why an executive order to wear masks?” 2020. https://www.facebook.com/110690805616809/posts/why-an-executive-order-to-wear-masks-the-problem-of-affect-in-political-terms-is/3202543303098195/

[20] *Gweilo Rant* “COVID 19: The Dangers of This Face Mask Frenzy; 2020. https://gweilorant.home.blog/2020/02/05/china-2019-ncov-the-dangers-of-this-face-mask-frenzy/0

[21] @themstems. Thanks Shane Neilson! Appreciate your help spreading the virus! Twitter; 2020.

[22] Fisher L . The illness experience: a feminist phenomenological perspective. In: Zeiler K , Käll LF , eds. Feminist Phenomenology and Medicine. Albany: SUNY Press; 2014:27‐46.

[23] Mercuri M . Just follow the science: A government response to a pandemic. J Eval Clin Pract. 2020;26(6):1575‐1578. 10.1111/jep.13491. 33043527 PMC7675691

